# A parameterized model for mean urinary inflow rate and its preliminary application in radiotherapy for cervical cancer

**DOI:** 10.1038/s41598-017-00356-9

**Published:** 2017-03-21

**Authors:** Fu Jin, Huan-Li Luo, Juan Zhou, Ding-Yi Yang, Li Yin, Xiao-Qing Yang, Ya-Nan He, Xian-Feng Liu, Da Qiu, Ming-Song Zhong, Han Yang, Chao Li, Qi-Cheng Li, Guang-Lei He, Ying Wang

**Affiliations:** 1 Division of Physics, Chongqing Cancer Institute & Hospital & Cancer Center, Chongqing, People’s Republic of China; 2 Department of Radiation Oncology, Chongqing Cancer Institute & Hospital & Cancer Center, Chongqing, People’s Republic of China; 3 Forensic Identification Center, College of Criminal Investigation, Southwest University of Political Science and Law, Chongqing, People’s Republic of China; 4 Medical Information and Engineering School, Southwest Medical University, Luzhou, People’s Republic of China

## Abstract

Forty-nine patients with stage II_b_ cervical cancer were included to investigate the changes in bladder volume in response to different approaches to maintaining consistent bladder filling. The impacts of age (*P*_age_), water consumption (*P*_*wat*_), and body mass index (BMI, *P*_*bmi*_) on the mean urinary inflow rate (*v*_*tot*_) were analysed. The bladder volume (BV) increased linearly over time. A large variation in *v*_*tot*_ among individuals was observed, ranging from 0.19 to 5.13 ml/min. The *v*_*tot*_ was correlated with *P*_age_ (*R* = −0.53, *p* = 0.01) and *P*_*wat*_ (*R* = 0.84, *p* = 0.00), and no correlation between *v*_*tot*_ and *P*_*bmi*_ was found (*p* > 0.05). Therefore, *v*_*tot*_ could be parameterized using two methods: multivariable linear regression and iterative fitting. There was no statistically significant difference between the two methods. The model accuracy was successfully assessed with several validation tests for patients with good compliance (79.2% of all patients), and the proportion of radiotherapy (RT) fractions with zero wait time (one ultrasound (US) scan) increased from 6.5% to 41.2%. The optimal US scanning number and RT time could be provided using this model. This adaptive RT approach could reduce patient discomfort caused by holding onto urine and reduce technician labour as well as cost.

Highly conformal radiotherapy (RT) techniques for cervical cancer enable adequate dose coverage of the clinical target volume (CTV) and the sparing of normal tissue^1, 2^. However, large inter-fraction organ motion of the cervix and uterus increases the risk of under-dosing the CTV or exposing the small bowel to a high dose of radiation. Several studies have demonstrated that the cervix and uterus move considerably due to tumour regression and variations in bladder and rectal filling^3–5^ and that uterine motion is greater than cervical motion. Uterine motion is predominantly influenced by bladder filling, with uterine fundus displacement of up to 48 mm^6^, and by cervical motion via rectal filling^7^.

RT with a full bladder is usually preferred because of the greater sparing of the small bowel^8^. However, it has been demonstrated that even with detailed instruction, when drinking a fixed volume of water at a fixed time (30–60 min) prior to each fraction, patients were unable to maintain consistent bladder filling^9, 10^. The use of cone-beam computed tomography (CBCT) scans enabled more accurate target definition and more precise tumour localization during radiation^11^, but the improvement was not sufficient to handle all the intra- and inter-fraction variabilities. An inherent drawback is the delayed bladder volume (BV) value. Following CBCT, the radiation oncologist must contour the entire outer surface of the bladder quickly and calculate the BV value to test its consistency. As a result, patient waiting time is inevitably prolonged. In addition, the time constraint is an important factor. More time is required for the execution of CBCT for each fraction^12, 13^. Time efficiency is crucial for RT centres where demand is high. The cost of CBCT is a further drawback.

Recently, Luo reported that a consistent and reproducible BV could be acquired using a portable bladder scanner. It was concluded that the CTV-to-PTV (PTV: planning target volume) margin was reduced from 11.1 to 6.4 mm in the superior–inferior direction^14, 15^. However, multiple measurements might be required until the BV is consistent with that of the planning computed tomography (CT). This possibility raised concerns regarding patient comfort, additional labour for the technician, and increases in waiting time and cost.

The aim of this study was to propose a model that predicted the likely BV and to reduce the number of pre-treatment ultrasound (US) measurements. The model could be incorporated into the current protocol of image-guided radiotherapy (IGRT). By applying the enhanced protocol, we could achieve more consistent BVs and reduce patient pain and discomfort (due to holding the urine for a long time). In addition, several validation tests assessed the model accuracy based on US measurements of variable bladder filling. The possibility of a reduction in workload and patient burden were also analysed by examining an appropriate number of US measurements.

## Materials and Methods

### Patients

Forty-nine patients with stage II_b_ cervical cancer but without angiocardiopathy or diabetes were included. Ethics approval for this study was obtained from Chongqing Cancer Hospital’s ethics committee, and screening was performed. All patients gave written informed consent. All methods were performed in accordance with the relevant guidelines and regulations. The patients’ mean age was 55.3 yr (median, 55 yr; range, 40–75 yr), and their mean BMI was 23.0 kg/m^2^ (median, 23.2 kg/m^2^; range, 17.6–28.2 kg/m^2^). The mean daily water consumption was 319 ml (median, 300 ml; range, 60–600 ml). Definitive RT was performed consecutively on all patients. The patients were randomly divided into two groups: a model group (25 patients) and a test group (24 patients). The datasets in the model group were collected to establish a parameterized model, and those in the test group were used to validate this model. Patients in both groups were asked to empty their bladders and then underwent controlled water intake prior to the planning CT and each subsequent RT fraction. Water intake prior to each fraction depended on the patient’s initial BV at the time of the planning CT.

### Bladder scan protocol

The BV measurements were made with a portable three-dimensional ultrasound bladder scanner (BVI9400, Verathon Medical B.V., Europe) for all patients. Then, on the CT day, a CT scan was performed. US measurements were taken throughout the treatment period, followed by pre-RT CBCT and post-RT bladder voiding. The post-void residual urine was also evaluated. The times at which the planning CT scan, US measurement, CBCT, and urination occurred were recorded.

For each patient in the model group, the first BV measurement occurred one hour after the patient drank the required amount of water but before each fraction was taken. Thereafter, multiple measurements might be required at regular 10-min intervals until the BV was consistent with that of the planning CT. Based on a previous report by our group, the default tolerance of BV is recommended to be within ±15% to maintain a constant BV^14–16^. This value might be updated after dosimetry data supporting the clinical effects are analysed.

In the test group, the first US measurement was taken at the predicted time based on the parameterized model. If the BV was not consistent with the expected value, which depended on the parameterized model with a correction factor, the patient was instructed to void his or her bladder or to wait for another measurement.

### A parameterized model for mean urinary inflow rate

A parameterized model was developed based on several assumptions:

1. The BV increases linearly over time, and the mean urinary inflow rate (*v*_*tot*_) is constant for each patient;
2. *V*_*tot*_ is different for different patients;
3. *V*_*tot*_ is affected mainly by three independent variables, namely, age (*P*_*age*_), water consumption (*P*_*wat*_), and body mass index (*P*_*bmi*_);
4. The correlation between *v*_*tot*_ and *P*_*age*_, *P*_*wat*_ or *P*_*bmi*_ is linear, and *v*_*tot*_ is a linear superposition of these functions. Thus,

1 $${v}_{tot}={v}_{0}+{v}_{age}+{v}_{wat}+{v}_{bmi};$$

2 $${v}_{age}={k}_{age}\ast {P}_{age};$$

3 $${v}_{wat}={k}_{wat}\ast {P}_{wat};$$

4 $${v}_{bmi}={k}_{bmi}\ast {P}_{bmi},$$

where *v*_*0*_ is a constant, called the net urinary inflow rate; *v*_*age*_, *v*_*wat*_, and *v*_*bmi*_ are the corresponding partial inflow rates affected by age, water intake, and body mass index, respectively; and *k*_*age*_, *k*_*wat*_, and *k*_*bmi*_ are the coefficients.

### Data analysis

The entire outer surface of the bladder was contoured on the axial slices using CT or CBCT images by experienced radiologists and radiation oncologists. Statistical analyses were performed using the Statistical Package of Social Sciences (IBM SPSS statistics version 22, SPSS22) program, where the threshold for statistical significance was p < 0.05. Due to the Gaussian distribution of the BV variation, the linearity of the time trend of BV and the correlation between *v*_*tot*_ and *P*_*age*_, *P*_*wat*_ or *P*_*bmi*_ were tested using Pearson’s coefficient. The relationships among these variables were tested by performing multivariable linear regression with SPSS22. In addition, an iterative fitting method was proposed to double-check these correlations. A detailed description of this analysis is presented in the next section. The difference in the mean urinary inflow rate between the two methods was analysed using paired t-tests.

### An iterative fitting method

In an investigation of the parameters that influence the urinary inflow rate, Heidi *et al*. showed that the impact of body mass index (*P*_*bmi*_) on the urinary inflow rate was negligible^17^. Hence, *k*_*bmi*_ = 0 and Equation 1 were simplified to only two arguments, *P*_*wat*_ and *P*_*age*_, and one constant, *v*_*0*_. In the model group, 4 patients had the same *P*_*wat*_ value. Using the datasets of these patients, *k*_*wat*_ was assigned a specific value, and Equation 1 was simplified again to only one argument, *P*_*age*_. The mean value of *k*_*age*_ was derived from the above four patients as an initial value of *k*_*age*_ (*k*_*age, ini*_) to calculate the initial *v*_*age*_, while the rest of the equation, (*v*_*tot*_ − *k*_*age, ini*_ * *P*_*age*_), was set as the initial value of (*v*_*0*_ + *v*_*wat*_). The correlations of initial *v*_*age*_ vs. *P*_*age*_ and initial (*v*_*0*_ + *v*_*wat*_) vs. *P*_*wat*_ were derived simultaneously from linear fitting. The fitted parameters were considered the new values of *k*_*age*_ and *k*_*wat*_, and they were used to calculate the new *v*_*age*_ and (*v*_*0*_ + *v*_*wat*_). The new *v*_*age*_ vs. *P*_*age*_ and the initial (*v*_*0*_ + *v*_*wat*_) vs. *P*_*wat*_ were fit. These loops were repeated until the difference between the fitted parameter and the two consecutive loops was less than 0.01. In addition, the correlation between *v*_*bmi*_ vs. *P*_*bmi*_ was tested using linear fitting.

## Results

### Time trend of bladder volume

The relative BV was defined as

5 $$\Delta V(t)=V(t)-{V}_{0},$$

and the relative deviation of the BV was defined as *ΔV*(*t*)/*V*_*0*_, where t is the time of the measurement. *V*_*0*_ was the BV from the first US scan (t = 0 min), and *V*(*t*) was the measured BV at regular 10-min intervals until BV consistency was reached. *ΔV*(*t*) was the corresponding difference.

**Figure 1:**
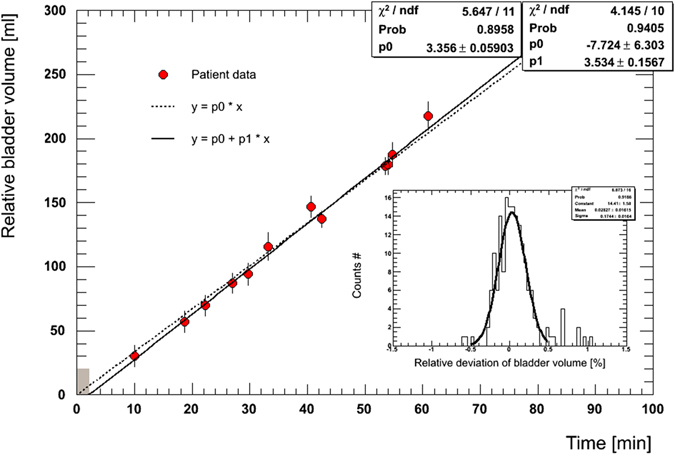
Typical example of the urinary inflow together with the linear fit. The urinary inflow rate was calculated as the slope of the linear fit line.

The relative BV as a function of time showed a linear behaviour for all 25 patients (p < 0.01). Figure 1 shows the BV as a function of time for one individual (*R* = 0.996, *p* = 0.00), and the error bars indicate the standard deviation. Due to the pre-RT variable waiting times and the repeated US measurements, the positions of the data points in Fig. 1 are around the predefined time measurements (i.e.,10, 20, 30, 40, 50 and 60 min). Two types of linear fitting were performed: 1) no uncertainty in scanning time, *v*_*tot*_ = 3.356 ± 0.060 ml/min with *χ*2 (the goodness of the fit)/degrees of freedom (*ndf*) = 5.647/11 (probability, *prob* = 0.896); 2) maximum uncertainty in time due to multi-scanning, *v*_*tot*_ = 3.534 ± 0.157 ml/min (*χ* ^*2*^ */ndf* = 4.145/10, *prob* = 0.940). The width of the brown box in Fig. 1 represents the maximum uncertainty of 2.18 min. The sub-panel in Fig. 1 shows the corresponding relative deviation of the BV, and Gaussian fitting was performed with *χ* ^*2*^ */ndf* = 8.873/16 (*prob* = 0.918).

### A parameterized model

The *v*_*tot*_ was correlated with *P*_*age*_ (*R* = −0.53, *p* = 0.01) and *P*_*wat*_ (*R* = 0.84, *p* = 0.00), and no relation between *v*_*tot*_ and *P*_*bmi*_ was found (*p* > 0.05).

### A multivariable linear regression method

The relations between *v*_*tot*_ and *P*_*age*_, *P*_*wat*_ or *P*_*bmi*_ could be expressed as follows:

6 $${v}_{{0}}=3.196\pm 2.410;(p=0.199)$$

7 $${v}_{age}=(-0.047\pm 0.017)\ast {P}_{age};(p=0.013)$$

8 $${v}_{wat}=(0.007\pm 0.001)\ast {P}_{wat};(p=0.000)$$

9 $${v}_{bmi}=(-0.003\pm 0.073)\ast {P}_{bmi}(p=0.970)$$

10 $$\begin{matrix}{v}_{tot}=3.196(\pm 2.410)-0.047(\pm 0.017){P}_{age}+0.007(\pm 0.001){P}_{wat}\\ \quad \quad -0.003(\pm 0.073){P}_{bmi};(p=0.00)\end{matrix}$$

The impact of *P*_*bmi*_ on *v*_*tot*_ was negligible and was verified once again in Equation 9. Thus, only two variables, *P*_*age*_ and *P*_*wat*_, were used to represent *v*_*tot*_.

11 $${v}_{0}=3.115\pm 1.128;(p=0.011)$$

12 $${v}_{age}=(-0.047\pm 0.016)\ast {P}_{age};(p=0.010)$$

13 $${v}_{wat}=(0.007\pm 0.001)\ast {P}_{wat};(p=0.000)$$

14 $${v}_{tot}=3.115(\pm 1.128)-0.047(\pm 0.016){P}_{age}+0.007(\pm 0.001){P}_{wat};(p=0.00)$$

### An iterative fitting method

**Figure 2:**
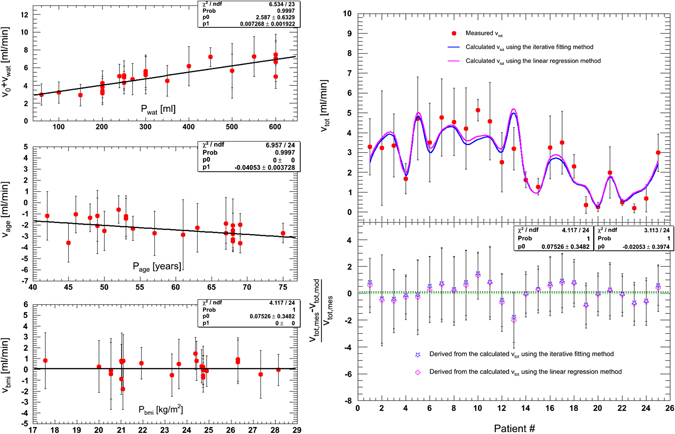
The left panel: *v*_*bm*_ *i* as a function of BMI in kg/m^2^ together with linear fitting (bottom); *v*_*age*_ as a function of age in years together with linear fitting (middle); (*v*_*0*_ + *v*_*wat*_) as a function of water intake in millilitres together with linear fitting (top). The right panel: The mean urinary inflow rates of all patients, including the measured value and the calculated value using two methods (top); the difference between the measured value and the calculated value (bottom).

The optimal solutions of Equation 1 (following several loops) are shown on the left-side of Fig. 2. The top panel of Fig. 2 shows (*v*_*0*_ + *v*_*wat*_) as a function of water intake in millilitres, and the middle panel shows *v*_*age*_ as a function of age in years. *V*_*bmi*_ was plotted as a function of *P*_*bmi*_, as shown in the bottom panel of Fig. 2. Linear fitting was performed. The values of *k*_*wat*_, *k*_*age*_ or *k*_*bmi*_ are the slope of the corresponding linear fitting lines, and *v*_*0*_ is the ordinate at the origin; thus,

15 $${v}_{{0}}=2.587\pm 0.633;$$

16 $${v}_{wat}=(0.007\pm 0.002)\ast {P}_{wat};$$

17 $${v}_{age}=(-0.040\pm 0.004)\ast {P}_{age};$$

18 $${v}_{bmi}=(-0.000\pm 0.000)\ast {P}_{bmi};$$

19 $${v}_{tot}=2.587(\pm 0.633)-0.040(\pm 0.004){P}_{age}+0.007(\pm 0.002){P}_{wat}.$$

All linear fits had high probabilities ((*v*_*0*_ + *v*_*wat*_): *prob* = 0.9997; *v*_*age*_: *prob* = 0.9997; *v*_*bmi*_: *prob* = 1).

The urinary inflow rates for the 25 patients are shown in Fig. 2 (right, top). The red points are the mean inflow rates for the individuals, and the error bars indicate standard deviations. Despite the provided drinking instructions, there was large inter-patient variation in the inflow rate (0.19 to 5.13 ml/min). No significant difference was observed between the calculated inflow rates using the two methods (blue line: the fitting method; pink line: the regression method). The difference between the measured and the calculated *v*_*tot*_ is shown in the lower right panel of Fig. 2. Two linear fits were used to obtain the mean relative deviation of *v*_*tot*_ (the fitting method: 0.07 ± 0.34; the regression method: −0.02 ± 0.40). Therefore, the maximum relative deviation of 0.42 was used as the tolerance in the validation test of this parameterized model.

### The validation test of this model

**Figure 3:**
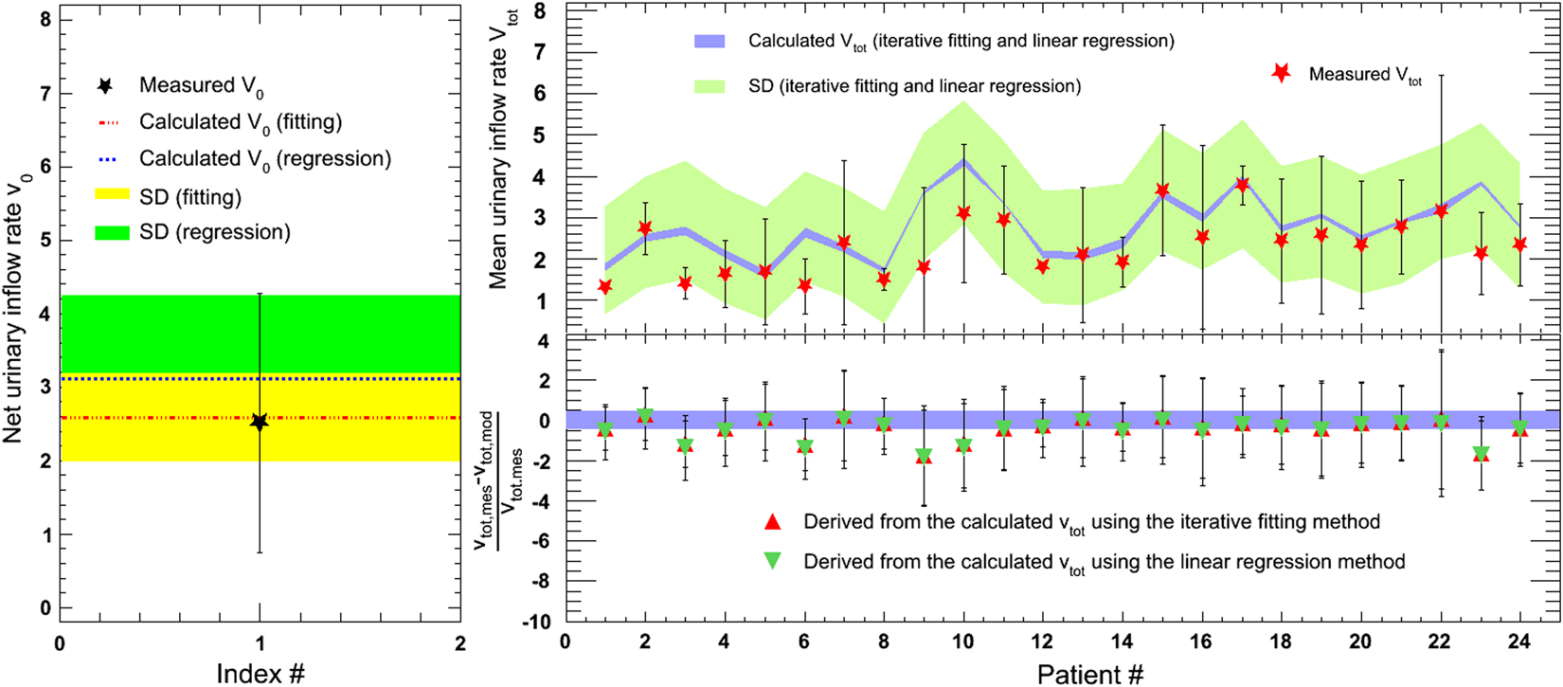
The comparison of net urinary inflow rates in the test group between measured and calculated values (left); the mean urinary inflow rates of all patients in ml/min in the test group, including the measured value and the calculated value using two methods (right, top) and the difference between two methods (right, bottom); SD: standard deviation.

Through US measurements taken in the test group 0 and 10 min after emptying the bladder before drinking water, two BV values were obtained, and the net urinary inflow rate, *v*_*0*_, was calculated using the expression (BV(10 min) −BV(0 min))/10. The mean of all 24 measured values was 2.51 ± 1.76 ml/min (black star in the left panel of Fig. 3). The calculated *v*_*0*_ was 2.59 ± 0.63 ml/min (dash-dot line plus yellow band, iterative fitting) or 3.12 ± 1.13 ml/min (dotted line plus green band, linear regression) using the parameterized model. These values were consistent with *v*_*0*_ within one standard deviation.

After plugging the values of *P*_*age*_ and *P*_*wat*_ into Equations 14 and 19 for all cases in the test group, the mean urinary inflow rate *v*_*tot*_ was obtained, as shown in the upper right panel of Fig. 3 (purple band plus yellow-green band). The bands represent the range of the calculated *v*_*tot*_ using two methods, and the red stars are the measured *v*_*tot*_ using the US scanner. Only 5 cases showed substantial discrepancies, and the relative deviation between the calculated *v*_*tot*_ and the measured *v*_*tot*_ was larger than 0.42 (purple band), as seen in the lower right panel of Fig. 3. Nineteen patients (79.2% of all patients) showed a high degree of consistency (≤0.42).

### US scanning number and waiting time before RT

**Figure 4:**
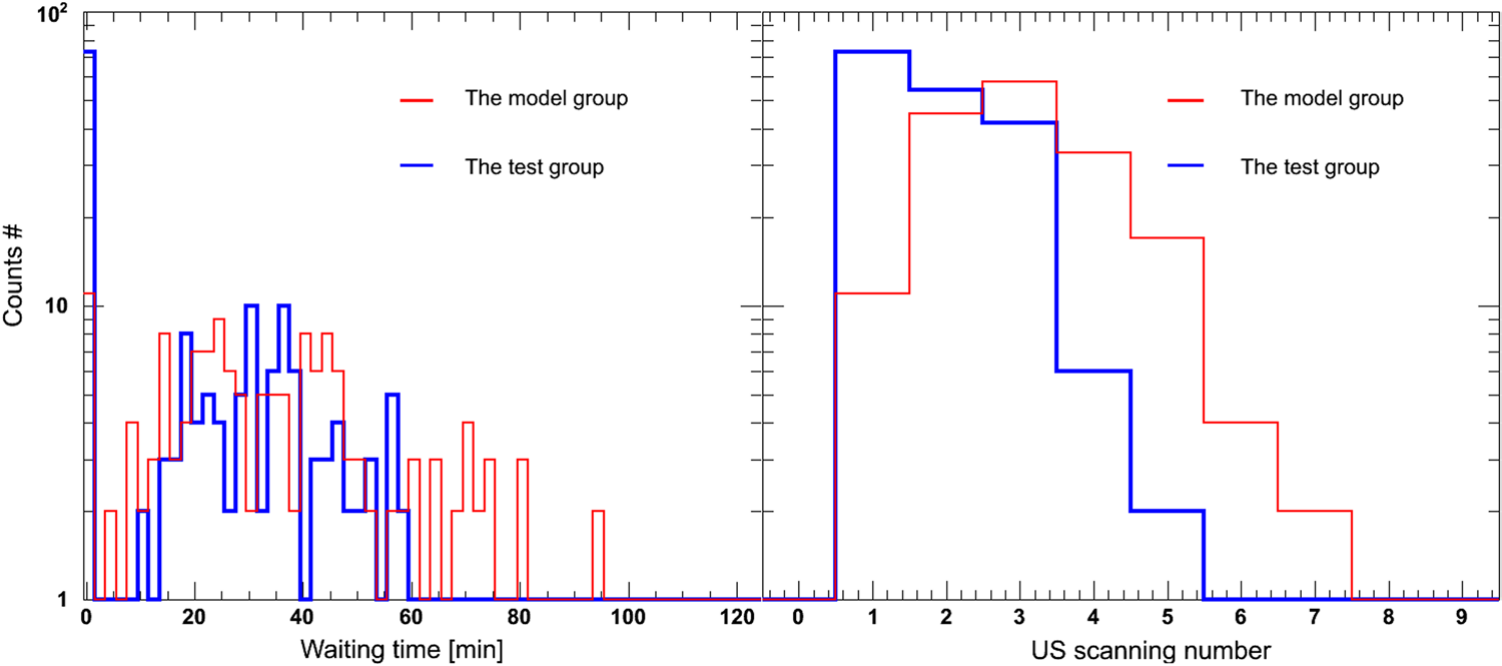
The histogram of waiting time and US scanning number of patients during RT fractions.

The waiting time and US scanning number before RT are shown in Fig. 4. In this work, the waiting time was defined as the amount of time that elapsed between one hour after drinking water (the model group) and the true RT or the predicted RT based on the parameterized model (the test group). In the model group, 6.5% of the patients had zero wait time for one US, while this percentage was 41.2% in the test group. In 71.2% of the RT fractions, the waiting time ranged from 12 min to 60 min in the model group, while this was the case for 54.2% of the cases in the test group. For the US scanning number, the peak value of the distribution was 1 for the test group (41.2%) and 3 for the model group (34.1%). All of the RT fractions achieved BV consistency within 5 US scans in the test group, but 3.5% of cases in the model group could not achieve the desired consistency.

## Discussion

In the previous literature, a portable ultrasonic instrument was adopted to evaluate bladder filling with a short measurement time. This instrument showed a high precision volume readout for standard bladder phantom and actual urine^15^. In this study, we used this US scanner to investigate the BV change and mean urinary inflow rate (*v*_*tot*_) in relation to age (*P*_age_), water consumption (*P*_*wat*_), and body mass index (*P*_*bmi*_). We found that *v*_*tot*_ was only correlated with *P*_*wat*_ and *P*_age_ (*R* = −0.53), which was consistent with Lotz’s report in which *v*_*tot*_ was negatively correlated with age (*R* = *−*0.50, *p* = 0.038)^17^. No relation between *P*_*bmi*_ and *v*_*tot*_ was found. Therefore, we proposed a model to predict *v*_*tot*_ and tried to provide a comfortable RT strategy for new patients with cervical cancer.

A patient’s respiration and level of stress, as well as technician proficiency, have been found to affect the accuracy of BV measurements. Multiple US scans (>5) were adopted to reduce this deviation in the current study. A US scan usually takes approximately 12 seconds. Therefore, a complete BV measurement for a patient might take approximately 1 min. When we calculated *v*_*tot*_, the mean measurement time value was used, and the maximum time uncertainty was approximately 30 seconds. In view of this uncertainty, we used two linear fitting methods, and the results from these two methods were consistent within one standard deviation. In fact, the true time uncertainty was far less than 2.18 min. The discrepancy between them was very small. Therefore, we obtained *v*_*tot*_ using the first method without uncertainty in the scanning time.

Figures 2 and 3 indicate a large intra-patient variation in the inflow rate (0.19 to 5.13 ml/min), but the net urinary inflow rate *v*_*0*_ was 2.59–3.12 ml/min. In Sabrina’s report, the bladder inflow rate was 3.0 ± 2.7 ml/min over all treatment fractions, agreeing with our results within one standard deviation. In addition, we found a significant correlation between the bladder inflow rate and the average length of the systematic cervix-uterus intra-fraction displacement^18^. The inflow rate might be used to predict the cervix-uterus position during treatment. In the future, we will focus on modelling the correlation between the inflow rate and the cervix-uterus position, which could help to design an automatic plan for cervical cancer.

In this study, the median pre-treatment water consumption was 300 ml and ranged from 60 to 600 ml. These values covered most of the water-drinking amounts. Figure 4 shows that the patients without model assistance achieved BV consistency in 6.5% of RT fractions one hour after water intake, which was higher than those at other time points. This finding might be one of reasons for the success of most commonly used instructions involving drinking 300, 500 or 600 ml of water 1 hour before RT^9, 18–20^. At the same time, we found that BV consistency was achieved in 77.6% of the fractions within 2 hours after water intake. This finding indirectly verified Sabrina’s instruction to drink 300 ml of water 2 hours pre-RT^18^. In the clinical practice, 79.2% of patients’ *v*_*tot*_ could be predicted accurately, and 41.2% of the treatment fractions could be performed successfully after one US scan with the parameterized model using the US scanner. However, in more than 50% treatments, the parameterized model should be modified due to changes in weather, treatment reactions, and the timing of each appointment during RT fraction.

Considering the above findings, we suggested that patients drink a specified amount of water until they had a comfortably full bladder after emptying their bladder. Then, the BV was measured at a specific time for each RT fraction, which depended on the *v*_*tot*_ (*v*_*tot, old*_) calculated by Equation 14 or 19. If the first measured BV was consistent with the value on the planning-CT day, RT could be delivered. If not, a new *v*_*tot*_ (*v*_*tot, new*_) was calculated using the BV at the specific time divided by the time interval. The difference between *v*_*tot, old*_ and *v*_*tot, new*_ was regarded as a correction factor to guide the next US scan until BV consistency was achieved. In our practice, 95.5% of fractional RT was performed within 3 US scans, as shown in Fig. 4.

Over the course of RT, a systematic reduction in mean BV was found^21^. A decrease in mean BVs from 156 to 88 cm^3^ between the first and last weeks of treatment was reported^3^. Some adaptive strategies with weekly magnetic resonance imaging (MRI) and daily CBCT were discussed in previous studies^16, 22^. James reported that all patients achieved clinically acceptable target coverage while maintaining organ-at-risk dose sparing after weekly re-planning^19^. Recently, Luo found that the mean BV during the course of RT was approximately equal to the initial BV with US-scanner assistance^15^.

In view of the results from this study and the above-noted previous reports and certain impact factors including the price of a US scan (approximately 1/13 CBCT cost; approximately 1/17 MRI cost) and technician labour, we propose a new IGRT protocol for cervical cancer involving daily US measurements and weekly CBCT and MRI scans based on this model. We only need one specialized technician to calculate the US scanning and RT time and measure BV in a clinical preparation room, while treatment technicians deliver RT and conduct the patient’s weekly imaging in order to perform an adaptive RT. This strategy could reduce the patient discomfort caused by holding onto the urine, as well as reduce technician labour and cost.
